# Contributive Role of TNF-α to L-DOPA-Induced Dyskinesia in a Unilateral 6-OHDA Lesion Model of Parkinson’s Disease

**DOI:** 10.3389/fphar.2020.617085

**Published:** 2021-01-11

**Authors:** Maurício dos Santos Pereira, Gabriel Henrique Dias Abreu, Jeremy Rocca, Sabah Hamadat, Rita Raisman-Vozari, Patrick Pierre Michel, Elaine Del Bel

**Affiliations:** ^1^ Department of Basic and Oral Biology, FORP, Campus USP, University of São Paulo, Ribeirão Preto, Brazil; ^2^ Department of Physiology, FMRP, Campus USP, University of São Paulo, Ribeirão Preto, Brazil; ^3^ USP, Center for Interdisciplinary Research on Applied Neurosciences (NAPNA), Brazil; ^4^ Paris Brain Institute, Inserm U 1127, CNRS UMR 7225, Sorbonne Université UM75, Paris, France

**Keywords:** astrocyte, cannabidiol, dopamine, glutamate, IL-1β, microglia, neuroinflammation, TNF-α

## Abstract

Our present objective was to better characterize the mechanisms that regulate striatal neuroinflammation in mice developing L-DOPA-induced dyskinesia (LID). For that, we used 6-hydroxydopamine (6-OHDA)-lesioned mice rendered dyskinetic by repeated intraperitoneal injections of 3,4-dihydroxyphenyl-L-alanine (L-DOPA) and quantified ensuing neuroinflammatory changes in the dopamine-denervated dorsal striatum. LID development was associated with a prominent astrocytic response, and a more moderate microglial cell reaction restricted to this striatal area. The glial response was associated with elevations in two pro-inflammatory cytokines, tumor necrosis factor-α (TNF-α) and interleukin-1β. Treatment with the phytocannabinoid cannabidiol and the transient receptor potential vanilloid-1 (TRPV-1) channel antagonist capsazepine diminished LID intensity and decreased TNF-α levels without impacting other inflammation markers. To possibly reproduce the neuroinflammatory component of LID, we exposed astrocyte and microglial cells in culture to candidate molecules that might operate as inflammatory cues during LID development, i.e., L-DOPA, dopamine, or glutamate. Neither L-DOPA nor dopamine produced an inflammatory response in glial cell cultures. However, glutamate enhanced TNF-α secretion and GFAP expression in astrocyte cultures and promoted Iba-1 expression in microglial cultures. Of interest, the antidyskinetic treatment with cannabidiol + capsazepine reduced TNF-α release in glutamate-activated astrocytes. TNF-α, on its own, promoted the synaptic release of glutamate in cortical neuronal cultures, whereas cannabidiol + capsazepine prevented this effect. Therefore, we may assume that the release of TNF-α by glutamate-activated astrocytes may contribute to LID by exacerbating corticostriatal glutamatergic inputs excitability and maintaining astrocytes in an activated state through a self-reinforcing mechanism.

## Introduction

L-DOPA-induced dyskinesia (LID) represents a common and severe motor complication after dopamine-replacement therapy in Parkinson's disease. LID manifests as uncontrollable and purposeless movements that develop after several years of L-DOPA therapy (Cenci and Konradi, 2010). LID remains a largely unmet need despite the recent approval of different amantadine-based medication forms for this condition (Perez-Lloret and Rascol, 2018).

The neural mechanisms underlying LID are complex and far from clear, although significant progress has been made in recent years. LID development results most probably from two mutually interacting factors, the striatal dopaminergic denervation and the pulsatile nature of orally delivered L-DOPA (Meissner et al., 2006; Cenci, 2014). As a result, the corticostriatal glutamatergic neurotransmission becomes progressively dysregulated, and maladaptive changes develop in medium spiny neurons of the dopamine-denervated striatum (Calon et al., 2003; Picconi et al., 2003; Robelet et al., 2004; Cenci, 2007; Rylander et al., 2009; Sgambato-Faure and Cenci, 2012). Such changes are characterized by extensive morphological and molecular aberrant readjustments, including supersensitivity of D1 dopamine receptors, which become abnormally activated by synaptic dopamine, leading to hyperactivation of cAMP-dependent signaling and downstream signaling events (Darmopil et al., 2009; Cenci, 2014).

Neuroinflammation, a central component of Parkinson’s disease pathology, is also thought to contribute actively to LID onset and perpetuation (Del-Bel et al., 2016). Experimental data collected over recent years have demonstrated that a reactive gliosis process takes place in the dorsal striatum of dyskinetic rats (Bortolanza et al., 2015a). Notably, the presence of reactive microglial cells and reactive astrocytes was reported in the dorsal striatum of dyskinetic rats while soluble pro-inflammatory mediators such as TNF-α and IL-1β were found to be elevated in the same striatal region (Barnum et al., 2008; Mulas et al., 2016). Other markers classically associated with inflammatory responses, such as the cyclooxygenase-2 (COX-2) enzyme and the inducible nitric oxide synthase (iNOS), have also been reported to be activated in preclinical models of LID (Bortolanza et al., 2015a; Bortolanza et al., 2015b).

In line with these observations, several groups reported that modulation of the inflammatory response could significantly improve the efficiency and tolerability of L-DOPA treatment in preclinical Parkinson’s disease models of LID (Barnum et al., 2008; Bortolanza et al., 2015a; Bortolanza et al., 2015b; Martinez et al., 2015), suggesting that neuroinflammation may represent a core component of the dyskinetic state. In particular, drugs with well-characterized anti-inflammatory activity, as the glucocorticoid corticosterone and the non-selective COX inhibitor ibuprofen have proved capable of largely attenuate dyskinesia (Teema et al., 2016). Similarly, two tetracycline derivatives doxycycline and COL-3 that possess anti-inflammatory properties, efficiently inhibited dyskinesia in L-DOPA-treated parkinsonian rats (Bortolanza et al., 2020). We also found that co-administration of the TRPV-1 ion channel antagonist capsazepine (CPZ) with cannabidiol (CBD), a cannabis derivative with well-reported anti-inflammatory properties (Napimoga et al., 2009; Campos et al., 2012; Mori et al., 2017; Sonego et al., 2018; Dos-Santos-Pereira et al., 2020) improves abnormal involuntary movements induced by L-DOPA in a hemiparkinsonian dyskinetic mouse model (Dos-Santos-Pereira et al., 2016; Junior et al., 2020). Coherent with these observations, abnormal involuntary movements were exacerbated by peripheral administration of the bacterial inflammogen lipopolysaccharide (LPS) in preclinical models of dyskinesia (Mulas et al., 2016). However, the nature of signals at the origin of inflammatory-type reactions in the dopamine-denervated striatum of dyskinetic mice remains elusive. Besides, it remains to be established how soluble factors released by glial cells interact with neuronal cells to promote LID manifestations.

In the present study, we aimed to further address the role of neuroinflammation in LID development by combining *in vitro* and *in vivo* approaches. Our specific aims were to 1) determine whether a treatment with CBD and CPZ that inhibits LID manifestation (Dos-Santos-Pereira et al., 2016) can also restrain inflammatory changes in the striatum of dyskinetic mice, 2) evaluate the impact of the same treatment in cell culture paradigms that model LID-related inflammatory mechanisms, and finally and above all 3) better characterize cellular and molecular mechanisms that contribute to LID development. Our results suggest that reactive astrocytes, the pro-inflammatory cytokine TNF-α and the neurotransmitter glutamate may represent critical elements in LID onset and perpetuation.

## Material and Methods

### Pharmacological and Cell Culture Reagents

6-hydroxydopamine hydrochloride (6-OHDA; #H4381, diluted in 0.9% saline), L-DOPA hydrochloride (#D1507, diluted in 0.9% saline), benserazide hydrochloride (#B7283, diluted in 0.9% saline), dopamine hydrochloride (DA; #H8502, diluted in distilled water), glutamate acid monosodium salt hydrate (Glu; #G5889 diluted in distilled water), capsazepine (CPZ; #C191, diluted in 50% DMSO-saline or 25% DMSO-distilled water), lipopolysaccharide (LPS; *Escherichia coli* strain O26:B6; #L8274, diluted in distilled water) and Tumor Necrosis Factor-α human (TNF-α, #T6674, diluted in distilled water) were all obtained from Sigma-Aldrich. Cannabidiol (CBD, distilled in 5% Tween 80-saline or 5% Tween 80-distilled water) of high purity grade (≥98%) was kindly donated by THCPharm (Germany). Dulbecco’s modified Eagle’s medium (DMEM), DMEM/F-12 nutrient mixture, the penicillin/streptomycin cocktail, and 0.05% Trypsin-EDTA solution were obtained from ThermoFisher Scientific (Saint Aubin, France). Fetal bovine serum (FCS) and horse serum (HS) were provided by Biowest LLC (Eurobio, Les Ulis, France).

### Use of Animals for *in vivo* and *in vitro* Studies

*In vivo* studies were performed with male adult C57⁄BL6 mice (FMRP-USP, Ribeirão Preto, Brazil; 18–25 g body weight). Mice were housed under a 12-h light/dark cycle with access to food and water *ad libitum*. All experiments were performed under the institutional approval of the Animal Care and Use Committee of Universidade de São Paulo (CEUA 026/2013), following the Brazilian Law n° 11.794/2008 and the Guide for the Care and Use of Laboratory Animals adapted from the National Institutes of Health.

For *in vitro* studies, we used either pregnant female C57BL/6J mouse or Wistar rats obtained from Janvier LABS (Le Genest St Isle, France). Animals were housed, handled, and cared for in strict accordance with the European Union Council Directives (2010/63/EU). Experimental protocols were approved by the Committee on the Ethics of Animal Experiments Charles Darwin no. 5. We used newborn pups (postnatal day 1) for both microglial and astrocyte cultures and Wistar rat embryos (embryonic day 15.5) for cortical cultures.

### *In vivo* Studies

#### Striatal Lesioning With 6-OHDA

Overall, eighty C57⁄BL6 mice were used for the current study. Stereotaxic surgical procedures for 6-OHDA lesioning were based on a protocol described earlier to study LID development in parkinsonian mice with partial striatal denervation (Dos-Santos-Pereira et al., 2016). Briefly, mice were anesthetized with 2,2,2-tribromoethanol (250 mg/kg, i.p.) before being positioned into a stereotaxic frame (David Kopf, model USA, 9:57). Stereotaxic coordinates for injection in the dorsal region of striatum were: anteroposterior (AP) = +0.5, mediolateral (ML) = ±2.3 and dorsoventral (DV) = −3.9 and −3.0 with respect to Bregma. 6-OHDA was diluted at 3.75 mg/ml in 0.9% saline containing 0.02% ascorbic acid to prevent the neurotoxin’s autoxidation, and mice were microinjected with a total volume of 2 µl/injection on two distinct injection sites, at a speed rate of 0.5 μl/min. The sham group received saline in the same conditions. At the end of the microinjection, the cannula was left in place for three additional minutes to prevent the injected solution's reflux. At the end of the surgical procedure, the animals were kept warm until full recovery from anesthesia before being returned to the vivarium, where they received amoxicillin (5 mg/ml orally) for five days.

#### Experimental Design of Mouse Treatments

Nineteen days after 6-OHDA lesion surgery, seventy-four animals were selected based on their response to the rotational apomorphine test (≥three contralateral rotations/min) (Iancu et al., 2005). Then, lesioned animals were divided into three experimental groups to have comparable mean values for contralateral rotations (i.e., dopaminergic denervation) in each subgroup for an unbiased assessment of test parameters. Note that the sham group presented no significant contralateral rotations.

The first set of experiments was performed to evaluate the intensity of the glial inflammatory reaction and the dopaminergic lesion in 6-OHDA-lesioned mice treated with L-DOPA. For that, forty-six mice were divided into 1) a sham-operated group (Sham), corresponding to mice that were injected with saline instead of 6-OHDA and sacrificed 21 days after stereotaxic injection, 2) a 6-OHDA-lesioned group (6-OHDA), corresponding to mice that were sacrificed 21 days after 6-OHDA lesioning, 3) a 6-OHDA + Veh-treated group (6-OHDA/Veh) corresponding to 6-OHDA lesioned mice that received daily i.p. injections of Veh between day 21 to day 42 post-lesioning, and 4) a 6-OHDA + L-DOPA-treated group (6-OHDA/L-DOPA) corresponding to 6-OHDA lesioned mice that received daily i.p. injections of L-DOPA (25 mg/kg) and benserazide (10 mg/kg) between day 21 to day 42 post-lesioning. These two last groups were also analyzed to evaluate abnormal involuntary movement manifestation on days 1, 7, 14, and 21 of treatment with Veh or L-DOPA.

**FIGURE 1:**
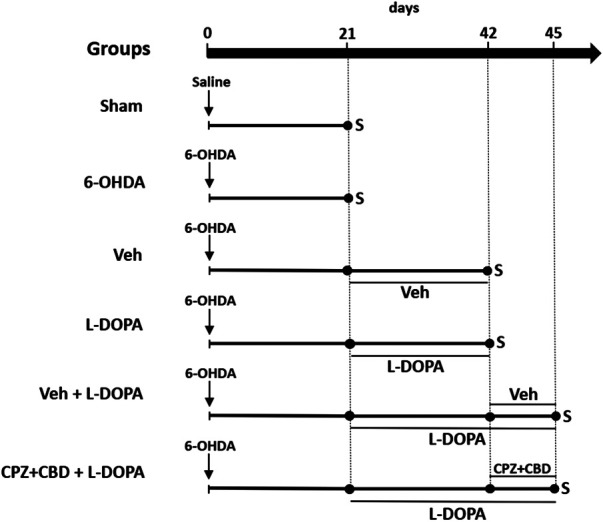
Schematic drawing summarizing the experimental design of *in vivo* experiments. Two groups of sham-operated and 6-OHDA-lesioned mice were sacrificed 21 days after stereotaxic surgery to assess the extent of nigrostriatal dopaminergic deficits and intensity of glial inflammatory changes in the striatum. Abnormal involuntary movements scores were also evaluated in the same groups before sacrifice. Two other groups of 6-OHDA-lesioned mice received daily i.p. injections of Veh or L-DOPA (25 mg/kg) + benserazide (10 mg/kg) from day 21 until day 42 to assess the same parameters as before and perform cytokine measurements. The two last groups of 6-OHDA-lesioned mice received L-DOPA in combination or not with CPZ (5 mg/kg i.p.) + CBD (30 mg/kg i.p.) from day 42 until day 45 to assess abnormal involuntary movements score and inflammation parameters. CPZ (5 mg/kg i.p.) and CBD (30 mg/kg i.p.) or corresponding Veh were given 30 and 15 min before L-DOPA, respectively or corresponding Veh. S: sacrifice.

The second set of experiments was performed to determine whether a treatment combining the TRPV-1 antagonist CPZ to the cannabinoid derivative CBD could modulate inflammation markers induced by L-DOPA in the striatum of dyskinetic mice. For that, twenty-eight animals divided into two experimental groups, referred to as 6-OHDA/L-DOPA + Veh or 6-OHDA/L-DOPA + CPZ + CBD, were treated according to experimental conditions described in a previous protocol (Dos-Santos-Pereira et al., 2016). Briefly, animals were treated with CPZ (5 mg/kg; i.p.; 30 min before L-DOPA) and CBD (30 mg/kg; i.p.; 15 min before L-DOPA) or with corresponding vehicles between day 43 and day 45 post-lesioning and analyzed on the third day of treatment to confirm LID reduction. Three hours after the last L-DOPA injection, mice from these two groups were sacrificed, and their brains rapidly dissected out for molecular analysis. Note that the treatment regimen with CPZ and CBD was selected based on previous data reporting on its efficacy against LID in our parkinsonian mouse model (Dos-Santos-Pereira et al., 2016). All treatment protocols are described in Figure 1.

#### Assessment of Abnormal Involuntary Movements

LID evaluation was performed by recording abnormal involuntary movements (Lundblad et al., 2005; Pavon et al., 2006; Dos-Santos-Pereira et al., 2016). Briefly, a trained observer evaluated abnormal involuntary movements in mice, 2, 30, 60, 90, 120, 150, and 180 min after L-DOPA administration. Axial, limb, and orolingual abnormal involuntary movements were recorded for 2 min and scored on a four-point scale according to their degree of manifestation using time (0 = not present; 1 = occasional; 2 = frequent; 3 = continuous but interrupted by sensory distraction, and; 4 = continuous) and intensity (0 = not present; 1 = weak; 2 = moderate; 3 = intense; and 4 = very intense) criteria. Cumulative scores are calculated as the sum of axial, limb, and orolingual scores at each time point over 3 h. A similar protocol was adopted for rotational behavior, i.e., circular movements toward the contralateral side to the lesion.

### *In vitro* Studies

#### Coating Procedures for Cell Cultures

The coating procedure was performed as described elsewhere (Dos-Santos-Pereira et al., 2018; Dos-Santos-Pereira et al., 2020). For astrocyte cultures, bottom surfaces of culture flasks were coated with 1 μg/ml laminin dissolved in sterile distilled water (Sigma Aldrich). For microglial and neuronal cell cultures, we used a borate buffer solution (pH 8.3) containing 1 mg/ml polyethyleneimine (PEI; Sigma Aldrich). After at least 2 h at 37 °C, culture flasks were washed with Dulbecco’s phosphate-buffered saline (PBS) four times before applying culture medium and cell seeding.

#### Microglial Cell Cultures

Microglial cell isolation was performed by taking advantage of microglia’s differential adhesion properties onto PEI coating under specific culture conditions (Sepulveda-Diaz et al., 2016). Briefly, newborn pups (postnatal day 1) were sacrificed, and the whole brain rapidly dissected out. The tissue was mechanically dissociated, and cells in suspension plated in PEI-coated T75 Corning culture flasks in the presence of DMEM supplemented with 10% FCS and 1% of a cocktail penicillin/streptomycin (Dos-Santos-Pereira et al., 2018; Dos-Santos-Pereira et al., 2020). The isolation was complete after about two weeks of culture. It was possible to maintain isolated microglial cells for one more week in culture flasks by adding small aliquots of DMEM supplemented with 1% FCS only. To produce subcultures, isolated microglial cells were trypsinized and then seeded onto uncoated Nunc 48 multi-well plates containing 500 µl a CSF-like N5 medium supplemented with 5% HS, 0.5% FCS, 5 mM glucose, and 100 μM glycine (Dos-Santos-Pereira et al., 2018; Dos-Santos-Pereira et al., 2020). Microglia were seeded at a density of 100,000 cells per well. Cultures were maintained at 37 °C under a humidified atmosphere of 95% air and 5% CO_2_. Experiments of stimulation were performed not later than 24 h after plating. These cultures were virtually free of astrocytes (<0.5%).

#### Astrocyte Cultures

To produce purified astrocyte cultures, we used a brain dissection protocol and tissue dissociation procedures similar to those described for microglial cells. The isolation of astrocytes required, however, different culture conditions. Specifically, cultured cells were grown using T75 Corning culture flasks coated with laminin and DMEM/F-12 nutrient mixture supplemented with 10% FCS and 1% of an antibiotic cocktail. In addition, cultured cells were treated twice a week with clodronate liposomes (3 μg/ml; Liposoma BV, Netherlands) to eliminate residual microglial cells. After two weeks, adherent astrocytes were dislodged mechanically from culture flasks and seeded at a density of 10^5^ cells in Nunc 48 multi-well plates coated with laminin and filled with 500 µl N5 medium supplemented as described for microglial cell cultures. Experiments of stimulation were performed in confluent astrocyte cultures, i.e., usually not later than three days after plating. These cultures were virtually free of Cd11b^+^ microglial cells (<0.1%).

#### Cortical Neuronal Cultures

Cortical cultures were prepared using rat Wistar embryos at day 15.5 of gestation using a protocol previously described (Lavaur et al., 2016). Dissociated cells in suspension were seeded at a density of 20,000–30,000 cells/well onto Nunc 48 multi-well multi-well plates pre-coated with 1 mg/ml PEI. These cultures were maintained in Neurobasal medium (Gibco, Saint Aubin, France) supplemented with a B27 cocktail without antioxidants (Gibco), an N2 mix (Gibco), and 100 IU/ml penicillin/streptomycin. These cultures also received 0.8 µM of ara-C, 2 h, and 18 h after plating. Under these conditions, cortical cultures contained only very few astrocytes and microglial cells. There was no need for culture medium renewal until the end of the experimental protocol. Experiments were carried out on mature cultures after 12 days of growth.

#### Cell Culture Treatment Protocols

As an attempt to mimic inflammatory processes in the striatum of mice developing LID, we established cultures of glial cells and exposed them to either dopamine, L-DOPA (3 and 10 µM), or glutamate (50 and 500 µM) for 24 h. LPS (0.5 ng/ml) was also used as a reference inflammogen treatment in the current setting. Cultured cortical neurons were stimulated with TNF-α (50 ng/ml) (Ye et al., 2013) or a reference treatment comprising 4-aminopyridine (4AP; 2.5 mM) and bicuculline (BIC; 50 μM) (Hardingham et al., 2002) in order to activate glutamate release. Treatments combining CPZ + CBD (each at 0.1 µM) were initiated 2 h before stimulation with the other treatments in either glial or neuronal cultures. Each drug concentration was selected based on preliminary experiments used to test the efficacy of the drug combination against LPS (10 ng/ml)-induced inflammation in either astrocyte or microglial cell cultures (not shown). Pharmacological treatments were either applied once for a 1-day treatment or renewed daily in case of prolonged treatment.

### Cellular and Molecular Analyses

#### Immunohistochemistry

Three hours after termination of L-DOPA (or Veh) treatment, mice were sacrificed by cervical dislocation and decapitated. Standard immunohistochemistry procedures were then carried out on free-floating sections using tyrosine hydroxylase (TH, 1:2000; Pel Freez, Arkansas, USA), glial fibrillary acidic protein (GFAP, 1:1,000; Millipore, Darmstadt, Germany) or ionized calcium-binding adapter molecule 1 (Iba-1, 1:400; Wako, USA) antibodies diluted in 0.1 M PBS (pH 7.4), containing 0.15% Triton X-100. Primary antibodies were incubated for 24 h and revealed with corresponding secondary antibodies using standard avidin-biotin immunohistochemical procedures. Peroxidase reactions were developed with the chromogen diaminobenzidine. All of these procedures have been described elsewhere in detail (Dos-Santos-Pereira et al., 2016). Note that tissue sections from different mice groups were processed, concomitantly, for each protein marker to avoid methodological biases.

#### Quantification on Immunostained Tissue Sections

TH immunocytochemistry was used for evaluating the loss of *substantia nigra pars compacta* dopaminergic cell bodies and that of striatal dopaminergic nerve endings after 6-OHDA lesioning. Counts of TH^+^ cell bodies were performed according to protocols described before (Dos-Santos-Pereira et al., 2016), and results were expressed in TH^+^ neurons/0.5 mm^2^. To evaluate the loss of dopaminergic nerve endings and the intensity of the astrocyte reaction after 6-OHDA lesioning, we measured optical densities (OD) of the TH and GFAP immuno-signals within the dorsal striatum. Results were expressed in % of OD in sham-operated mice. For descriptive purposes, microglial cells were categorized according to morphological criteria previously established, and we measured the number of microglial reactive cells in the dorsal striatum as reported previously (Gomes et al., 2015). Results were expressed in Iba-1+ cells/0.5 mm^2^.

#### Western Immunoblotting Quantification

Cultured cells were processed for Western Immunoblotting analysis using a previously established protocol (Santa-Cecilia et al., 2016). Briefly, cells were recovered with M-PER buffer (Invitrogen) containing protease and phosphatase inhibitor cocktail (Invitrogen), and aliquots were resuspended for protein quantification with a Nanodrop 8,000 Spectrophotometer (Thermo Fisher Scientific). Then, protein extracts were resolved on a 4–12% SDS-PAGE gel and transferred to PVDF membranes. Membranes were then incubated with primary antibodies against GFAP (1:1,000, overnight; Wako), Iba-1 (1:500, overnight; Dako) and GAPDH (1:2000, 2 h; Sigma-Aldrich) and washed with Tris-buffered saline containing 0.1% Tween-20 before incubation with the rabbit secondary IRDye antibody (LI-COR Biosciences, Lincoln, NE, USA). Blots were imaged through the LI-COR Odyssey infrared imaging system (LI-COR Biosciences), and quantitative analysis was performed with the ImageJ software (dos Santos Pereira et al., 2015).

#### Cytokine Measurement

The sandwich ELISA method was used to detect cytokines (TNF-α, IL-1β) in the dorsal striatum of mice or the culture medium of glial cells. For animal samples, approximately 10 mg of the dorsal area of the striatum (AP: +0.62 to +0.5; ML: ±2.2 to ±2.4; DV: +3.0 to +3.5; relative to Bregma) were isolated using an adult mouse brain slicer matrix (World Precision Instruments, Inc.; Sarasota, USA). Tissue amount was equalized at a concentration of 50 mg/ml and homogenized in a lysis buffer containing 20 mM Tris-HCl pH 8.0, 137 mM NaCl, 10% glycerol together with protease (10% v/v; Sigma) and phosphatase (1 tablet/10 ml; Roche) inhibitors. After centrifugation at 4 °C (10000 rpm for 10 min), the supernatant was recovered and immediately frozen at −80 °C before further processing. Samples were further diluted (1:10) to reach a final amount of 5 mg/ELISA microplate well for cytokine measurement. For cell cultures, the medium was removed 24 h after initiation of drug stimulation and frozen for subsequent analysis, with no need for sample dilution. Test samples were measured according to the manufacturer’s instructions using a spectrophotometer SpectraMax M4 (Molecular Devices, Sunnyvale, CA). ELISA standard curves were generated using a four-parameter logistic curve model (GraphPad Prism 8, GraphPad Software).

#### Glutamate Assay

Glutamate was assayed using the Amplex Red Glutamic Acid/Glutamate Oxidase Kit (#A12221; Invitrogen) according to the manufacturer’s instructions. The quantification was carried out with 25 μl of culture medium, and the fluorescent reaction product resulting from glutamic acid oxidation was quantified using a SpectraMax M4 microplate reader (Molecular Devices, Sunnyvale, CA).

### Statistical Analysis

The Mann-Whitney test and the Kruskal–Wallis followed by Dunn’s test were used to evaluate differences in abnormal involuntary movements scores between two or more groups, respectively. Unpaired Student’s t-test and One-Way-ANOVA followed by the Bonferroni post-hoc test were used to evaluate other changes between two or more than two groups, respectively. For all experiments, the significance level was set at *p* < 0.05. Graphs were generated, and statistical analysis was performed using the software GraphPad Prism (version 8). Statistical values are presented in Supplementary Table.

## Results

### L-DOPA-Induced Dyskinesia Characterization in Mice With a Partial Unilateral Lesion of the Nigrostriatal Dopaminergic Pathway

**FIGURE 2:**
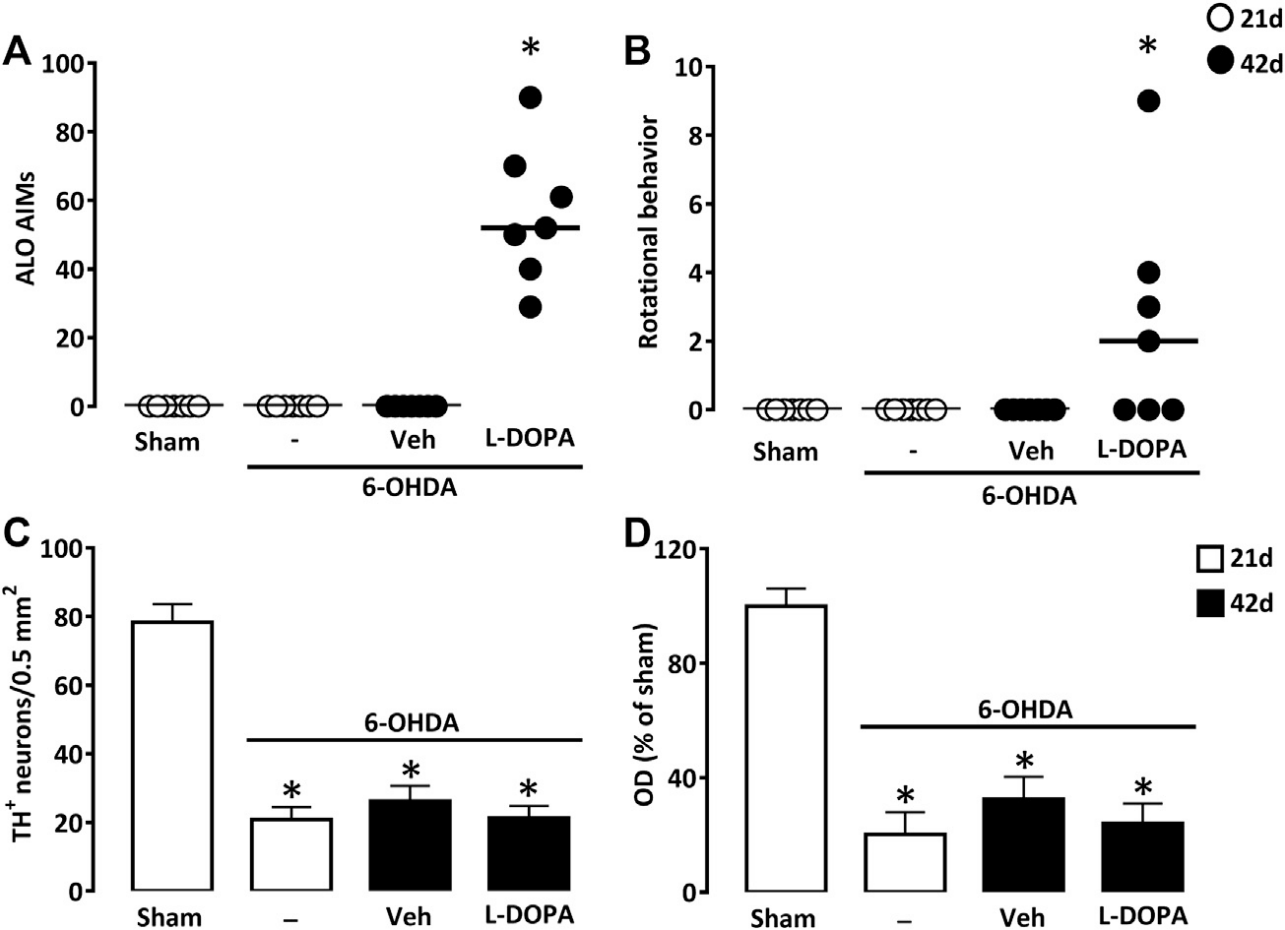
Abnormal involuntary movements after L-DOPA treatment in mice with partial unilateral lesions of the nigrostriatal pathway. **(A)** Sum of axial, limb and orofacial (ALO) abnormal involuntary movements (AIMs) of sham-operated and 6-OHDA-lesioned mice, 21 or 42 days after surgery. Between days 21–42, 6-OHDA-lesioned mice received daily injections of Veh or L-DOPA. **(B)** Sum of locomotor activity in the same groups of mice as in **(A)**. *p < 0.05 *vs.* sham, n = 7; Kruskal-Wallis analysis on ranks followed by Dunn’s test. **(C)** Density of TH^+^ cell bodies per 0.5 mm^2^ in the *substantia nigra pars compacta* and **(D)** Relative TH optical density (OD) in the striatum of the same groups of mice as in **(A)**. *p < 0.05 *vs.* sham, n = 7; One-way ANOVA followed by Bonferroni test. Data are presented as both median (horizontal black lines) and individual values (circles) in **(A)** and **(B)** and as mean ± SEM in **(C)** and **(D)**.

We performed a unilateral lesion of the nigrostriatal pathway with two successive injections of 7.5 µg 6-OHDA in two distinct sites of the dorsal striatal area. Daily treatment with L-DOPA between days 21 and 42 after surgery (6-OHDA/L-DOPA) resulted in severe axial, limb, and orofacial abnormal involuntary movements 42 days after 6-OHDA lesioning. The other groups of mice not receiving L-DOPA did not develop abnormal involuntary movements 21 (Sham, 6-OHDA) or 42 (6-OHDA/Veh) days after lesioning (Figure 2A). In 6-OHDA-lesioned mice, L-DOPA treatment also promoted rotational behavior characterized by a contralateral rotational behavior (Figure 2B).

TH immunodetection of *substantia nigra pars compacta* dopaminergic neurons revealed a robust but partial loss (−73.3%) of these neurons (Figure 2C). Measurements of OD in the dorsal striatum of TH immuno-positively stained tissue sections indicated that efferent dopaminergic nerve endings were reduced to a similar extent as nigral dopamine cell bodies (Figure 2D). Noticeably, nigral and striatal dopaminergic deficits were not different from those detected in the two other groups of lesioned mice treated with L-DOPA (6-OHDA/L-DOPA) or with vehicle (6-OHDA/Veh) that were sacrificed 42 days after stereotaxic surgery.

### Chronic L-DOPA Treatment Results in a Sustained Glial Inflammatory Response in the Dopamine-Denervated Mouse Striatum

**FIGURE 3:**
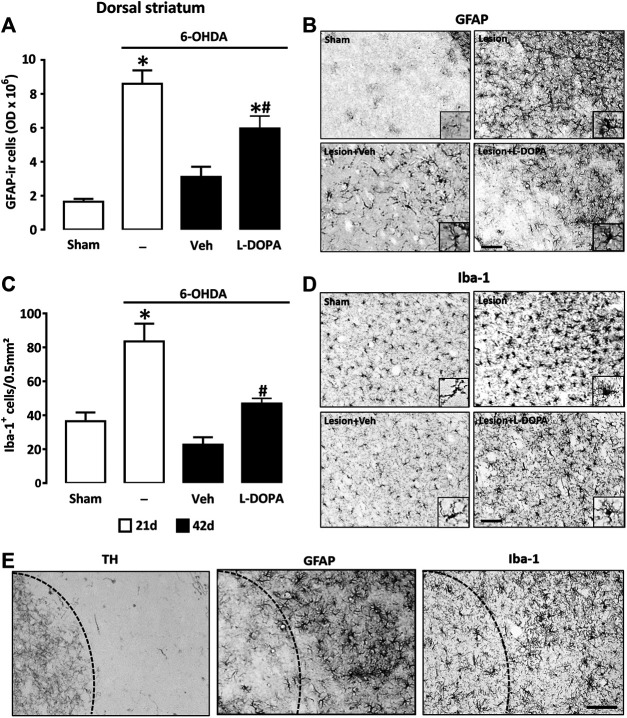
Quantification of the glial reaction in the dopamine-denervated dorsal striatum of mice receiving L-DOPA. **(A)** Intensity of the astroglial reaction evaluated by quantification of the GFAP immunosignal in the dorsal striatum of sham-operated and 6-OHDA-lesioned mice sacrificed at day 21 or at day 42 after stereotaxic surgery. Mice sacrificed at day 42 received Veh or L-DOPA treatment between days 21–42. *p < 0.05 *vs.* sham; #*p* < 0.05 *vs.* Veh, n = 7; One-way ANOVA followed by Bonferroni test. **(B)** Micrographs depicting GFAP^+^ cells in the dorsal area of the striatum of the same groups as before. Note profound changes in the number of GFAP^+^ astrocytes and in their morphology 21 days after 6-OHDA injection. At day 42, the astrocyte reaction is reduced but still intense in 6-OHDA-lesioned mice treated with L-DOPA. **(C)** Intensity of the microglial reaction was evaluated by quantifying the density of Iba-1^+^ cells in the dorsal striatum of the same groups as before. *p < 0.05 *vs.* sham; #*p* < 0.05 *vs.* Veh, n = 7; One-way ANOVA followed by Bonferroni test. **(D)** Micrographs depicting Iba-1^+^ cells in the dorsal area of the striatum of the same groups as before. Note that the microglial reaction is prominent in 6-OHDA-lesioned mice 21 days after stereotaxic surgery. At day 42 the microglial reaction is reduced but still present in L-DOPA-treated mice. **(E)** Immunocytochemical detection of TH, GFAP and Iba-1 in the dorsal striatum of 6-OHDA-lesioned mice treated with L-DOPA between day 21 and 42. Illustrations show that the glial reaction is restricted to the dorsal striatum where TH immunostaining is reduced. Dash lines represent the virtual boundary between the dorsal and ventral striatum. Micrographs for GFAP and Iba-1 are from slides at level +0.1 mm and +0.18 mm from Bregma, respectively. Scale bar: 100 µm.

To compare the astroglial and microglial cell responses in the denervated striatum of the three groups of 6-OHDA lesioned mice (6-OHDA, 6-OHDA/Veh, and 6-OHDA/L-DOPA), we performed immunohistochemical detection of GFAP and Iba-1, respectively. OD measurements of immuno-positively stained GFAP^+^ astrocytes in the dorsal striatal area revealed profound changes in this parameter between the four groups of mice (Figure 3A). Primarily, we established that the GFAP immunosignal was strongly increased 21 days post-lesioning (6-OHDA) in comparison to the sham-operated group (Sham), which indicates that the striatal injection of 6-OHDA provoked in itself a robust activation of astroglial cells. The GFAP immunosignal decreased but remained elevated in the L-DOPA-treated group (6-OHDA + L-DOPA), 42 days post-6-OHDA lesioning, whereas it returned to near control values in the group of mice treated with Veh (6-OHDA/Veh). This suggests that the inflammatory response of astrocytes, which regressed spontaneously overtime after 6-OHDA lesioning was partly maintained by L-DOPA treatment. Quantitative data are illustrated by representative micrographs of GFAP immunopositive cells from striatal coronal sections of each group of mice (Figure 3B). Correlated to OD measurements, we noted that the size of the astrocyte soma and the thickness of primary astrocyte processes were increased in 6-OHDA lesioned mice sacrificed 21 days post-lesioning (6-OHDA). Although partly attenuated in 6-OHDA lesioned mice given L-DOPA, these morphological changes were still observable until day 42 (6-OHDA/L-DOPA). However, they were absent in mice treated with Veh instead of L-DOPA (6-OHDA/Veh).

To evaluate the intensity of the microglial cell response, we quantified the number of Iba-1 immunoreactive cells in the striatum's dorsal area in the four experimental groups of mice. While the dorsal striatum of sham-operated mice (Sham) contained quiescent Iba-1^+^ microglial cells generally having only a few processes and small cell bodies (Figure 3C), that of 6-OHDA-lesioned mice (6-OHDA) was characterized by an increased number of Iba-1^+^ reactive cells with a high proportion of them having an amoeboid-like reactive morphology with short processes or a large soma with thick processes (Figure 3D). In the 6-OHDA lesioned group receiving L-DOPA (6-OHDA/L-DOPA), the number of reactive Iba-1^+^ cells was lower but remained elevated compared to the Veh-treated group (6-OHDA/Veh). Thus, similar to what is observed for astrocytes, the inflammatory response of microglial cells, which regressed spontaneously after 6-OHDA lesioning was partly maintained by L-DOPA treatment. At the cellular level, microglial cells also presented typical features of an activation state. At variance, the number of reactive Iba-1^+^ cells returned to below control values in the 6-OHDA group treated with Veh (6-OHDA/Veh) (Figure 3C,D). In these conditions, microglia presented a morphology characteristic of a resting state. It should be noted that the activation of astrocytes and microglial cells occurred almost exclusively in the dorsal area of the striatum, where the loss of TH^+^ nerve fibers is prominent (Figure 3E).

### The Pro-inflammatory Cytokines TNF-α and IL-1β Are Elevated in the Dopamine-Denervated Striatum of Dyskinetic Mice

**FIGURE 4:**
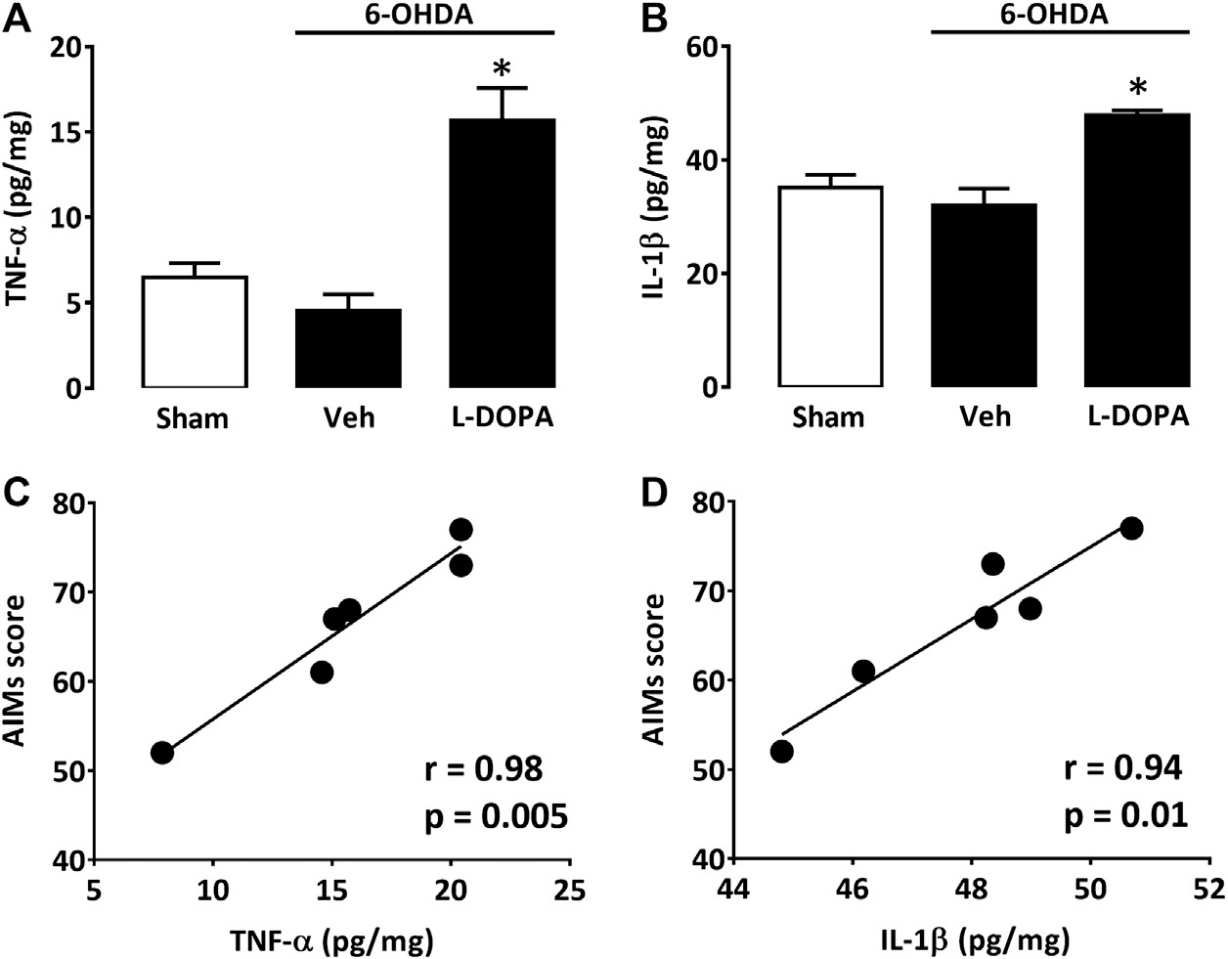
Cytokine levels in the dorsal striatum of L-DOPA dyskinetic mice. **(A)** TNF-α levels in sham-operated (21 days) and 6-OHDA-treated mice (42 days) receiving or not L-DOPA. **(B)** IL1-β levels in sham-operated (21 days) and 6-OHDA-treated mice (42 days) receiving or not L-DOPA. *p < 0.05 *vs.* sham, n = 6; One-way ANOVA followed by Bonferroni test. **(C)** Correlation between TNF-α values and abnormal involuntary movements scores in mice treated with L-DOPA. **(D)** Correlation between IL1-β values and abnormal involuntary movements scores in mice treated with L-DOPA. In **(C)** and **(D)** filled circles represent data values from individual animals. Spearman’s correlation coefficient and *p*-values are presented for each cytokine in the figure above.

Since astroglial and microglial cells were activated in the dorsal striatum of dyskinetic mice, we also wished to assess levels of two prototypical pro-inflammatory cytokines, TNF-α and IL-1β, within the same striatal area. TNF-α levels were low in sham-operated mice (Sham) and 6-OHDA-lesioned mice treated with Veh for 21 days after stereotaxic surgery (6-OHDA/Veh). Nevertheless, there was a substantial increase in TNF-α striatal levels in 6-OHDA-lesioned mice treated with L-DOPA (6-OHDA/L-DOPA) (Figure 4A). IL-1β was also significantly increased in L-DOPA-treated dyskinetic mice (6-OHDA/L-DOPA), although proportionally less than TNF-α (Figure 4B). Individual positive correlations were found between LID severity and TNF-α (Figure 4C) or IL-1β elevations (Figure 4D).

### The Antidyskinetic Treatment Combining Capsazepine + Cannabidiol Prevents the Production of TNF-α but Not That of IL-1β in the Dopamine-Denervated Striatum

**FIGURE 5:**
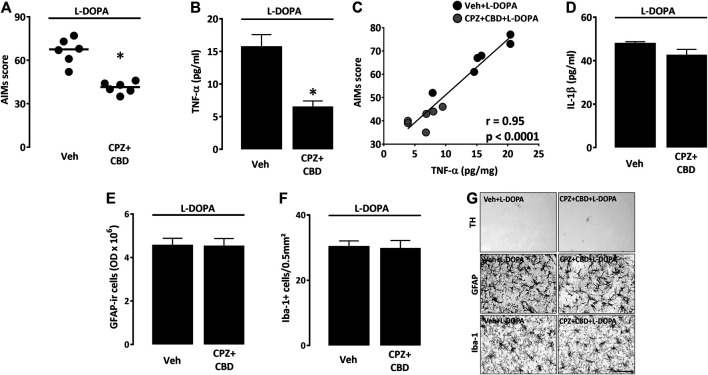
Impact of CPZ + CBD on LID manifestation and striatal inflammation parameters. **(A)** Impact of a treatment with CPZ (5 mg/kg i.p.) + CBD (30 mg/kg i.p.) on the abnormal involuntary movements (AIMs) score of dyskinetic mice receiving L-DOPA. Data are presented as median (horizontal lines) and individual values (filled circles). *p < 0.05 vs. Veh, n = 6; Mann-Whitney’s test. **(B)** Impact of CPZ + CBD on TNF-α levels within the dorsal injured striatum of dyskinetic mice. *p < 0.05 vs. Veh, n = 6; Mann-Whitney’s test. **(C)** Correlation plot between TNF-α values and AIMs scores in mice treated with Veh + L-DOPA or CPZ + CBD + L-DOPA. The Spearman correlation coefficient r and the *p* value are given above in the plot. **(D)** Absence of impact of CPZ + CBD on IL1-β levels within the dorsal injured striatum of dyskinetic mice. **(E)** Absence of impact of the CPZ + CBD treatment on the intensity of the GFAP immunosignal in the dorsal striatum of dyskinetic mice. **(F)** Absence of impact of the CPZ + CBD treatment on the number of Iba-1^+^ microglial cells in the dorsal striatum of dyskinetic mice. **(G)** Representative illustrations showing that the treatment with CPZ + CBD had no impact on the intensity of the GFAP immunosignal from astrocytes, the density of Iba-1^+^ microglial cells and the TH immunosignal of dopaminergic nerve endings in the dorsal striatal area of dyskinetic mice receiving L-DOPA. Scale bar: 50 µm.

We wished to determine whether the treatment with CPZ + CBD, reported being effective against dyskinesia (Dos-Santos-Pereira et al., 2016), also had the capacity of reducing levels of TNF-α and IL-1β in the dorsal striatum of 6-OHDA-lesioned mice treated with L-DOPA. As expected, the combined treatment with CPZ + CBD (6-OHDA/CPZ + CBD + L-DOPA) significantly attenuated axial, limb, and orofacial L-DOPA-induced abnormal involuntary movements (Figure 5A), without changing the rotational behavior of dyskinetic mice (data not shown). Most interestingly, we established that CPZ + CBD restrained the TNF-α elevation in L-DOPA-treated mice (6-OHDA/CPZ + CBD + L-DOPA) (Figure 5B). Note that there was a significant correlation between TNF-α levels and LID severity (Figure 5C). Surprisingly, the treatment with CPZ + CBD did not affect IL-1β levels in L-DOPA-treated mice (Figure 5D). Note that the treatment with CPZ + CBD had also no impact on the GFAP immunosignal from astrocytes and the density of Iba-1^+^ microglial cells in the dorsal striatal area of dyskinetic mice receiving L-DOPA (Figures 5E–G). This treatment was also without effect on striatal TH^+^ nerve endings in the same experimental conditions (Figure 5G).

### TNF-α Release Is Stimulated by Glutamate but Neither by L-DOPA nor Dopamine in Astrocyte Cultures

To determine why glial inflammatory markers were increased in the denervated striatum of dyskinetic mice, we established purified cultures of astrocytes and microglial cells and exposed them to candidate molecules that could mediate inflammatory processes in response to L-DOPA treatment in dyskinetic mice. Specifically, we tested the impact of dopamine, its precursor L-DOPA, and that of glutamate.

**FIGURE 6:**
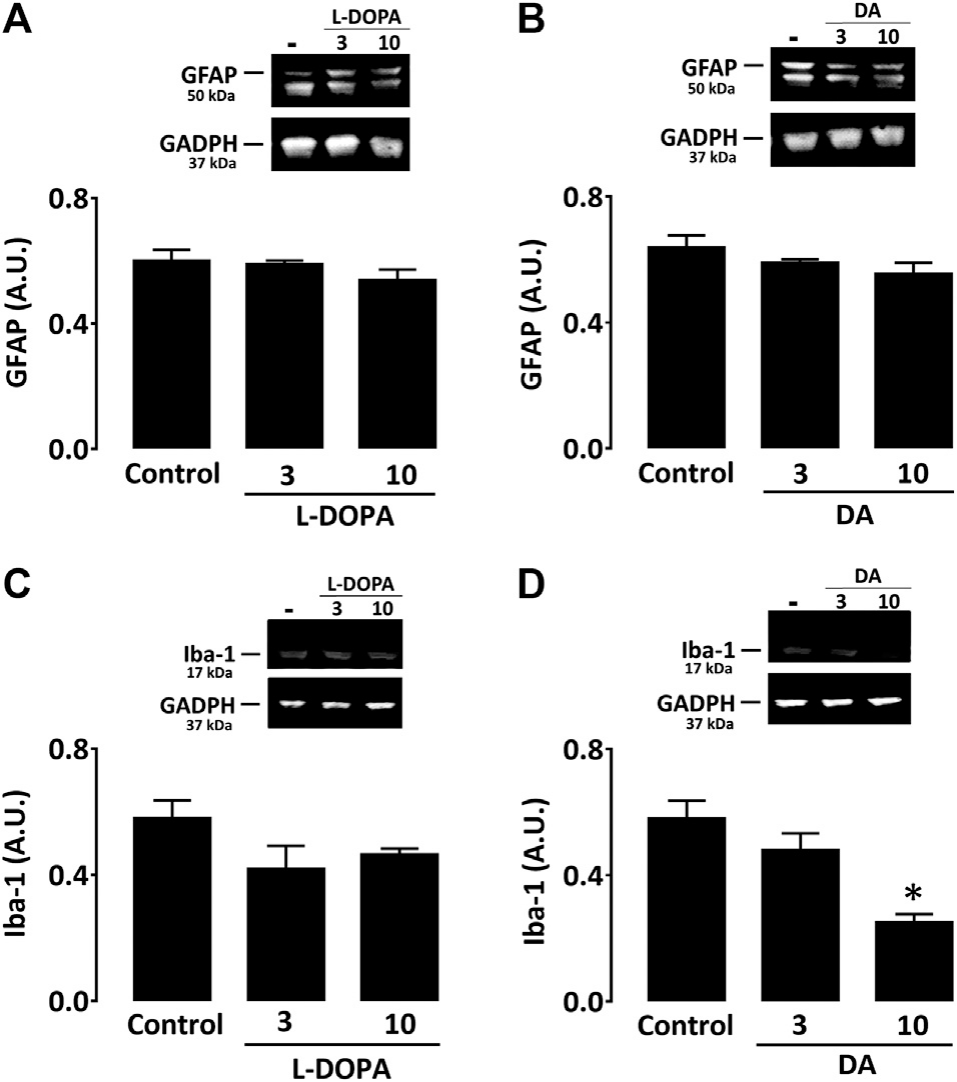
Neither L-DOPA nor dopamine promotes activation of astrocytes or microglial cells in culture. **(A)** Western immunoblotting characterization of GFAP expression in astrocyte cultures treated with L-DOPA (3 and 10 µM) or **(B)** dopamine (3 and 10 μM). **(C)** Western immunoblotting characterization of Iba-1 expression in microglial cultures treated with L-DOPA (3 and 10 µM) or **(D)** dopamine (3 and 10 μM). Note that 10 µM dopamine caused a significant decrease of the Iba-1 signal. *p < 0.05 *vs.* control, n = 5; One-way ANOVA followed by Bonferroni test. Data are expressed in arbitrary units (AU).

In cultured astrocytes, western immunoblotting quantification of GFAP expression revealed that neither treatment with L-DOPA nor dopamine (3 and 10 µM) had a significant impact on levels of this protein at two different concentrations (Figure 6A). In microglial cell cultures, western immunoblotting analysis showed that L-DOPA had no impact on Iba-1 expression levels at 3 and 10 µM, whereas dopamine induced a significant reduction of this inflammation marker at 10 µM (Figure 6C). L-DOPA and dopamine also failed to increase cytokine production in both types of glial cultures (not shown).

**FIGURE 7:**
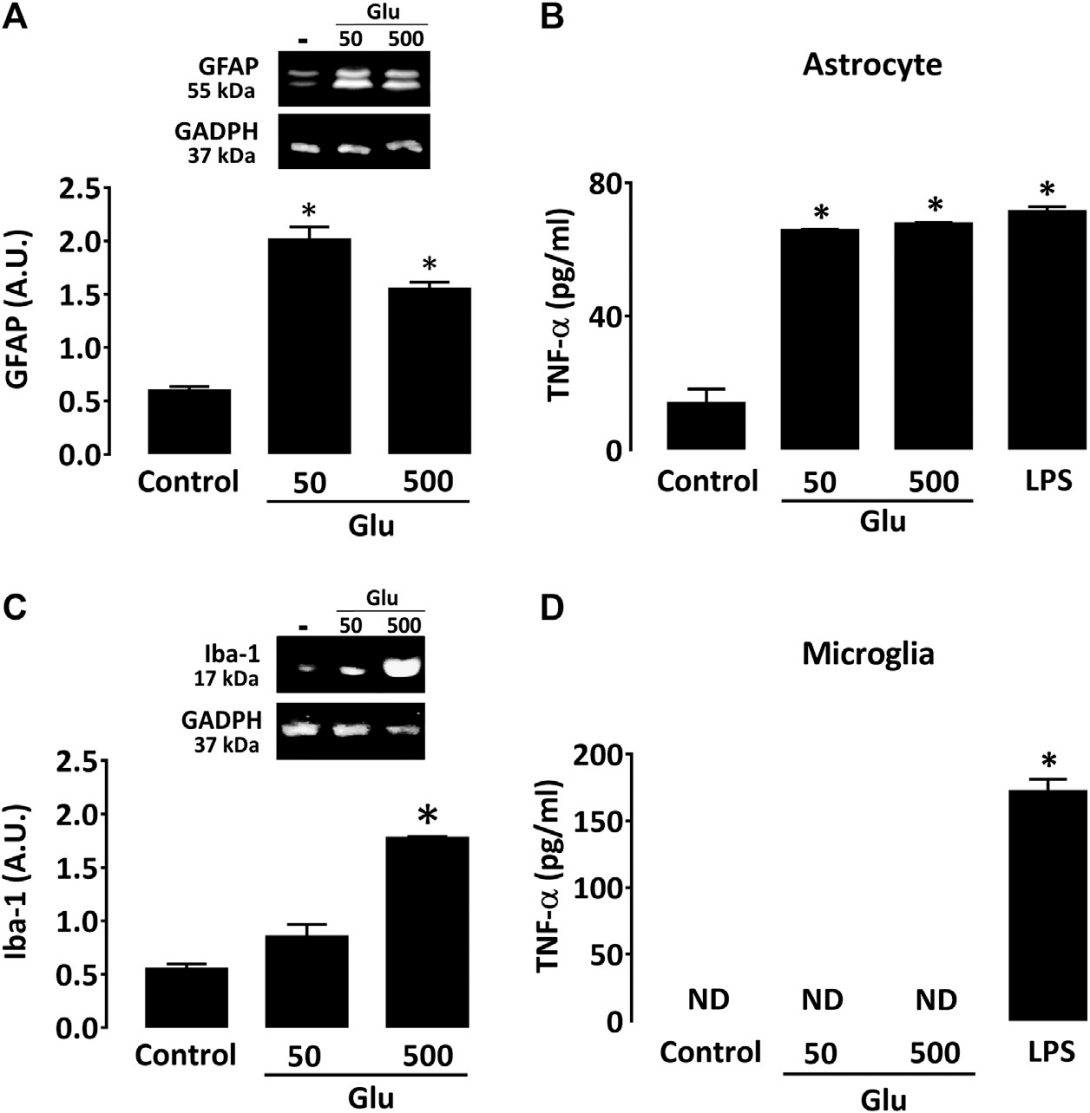
Glutamate promotes activation of cultured astrocytes. **(A)** Impact of glutamate (50 and 500 μM) on GFAP expression as characterized by western immunoblotting in astrocyte cultures. **(B)** Impact of glutamate (50 and 500 μM) on Iba-1 expression as characterized by western immunoblotting in microglial cultures. *p < 0.05 *vs.* control, n = 6; One-way ANOVA followed by Bonferroni test. **(C)** Impact of glutamate (50 and 500 µM) on TNF-α levels in astrocyte and **(D)** microglial cultures. In both cases, LPS (0.5 ng/ml) was used as reference inflammogen. *p < 0.05 *vs.* control, n = 6–8; One-way ANOVA followed by Bonferroni test. ND: Non-detectable.

When added to astrocyte cultures, glutamate caused a robust increase in GFAP expression (Figure 7A) at 50 and 500 µM. Glutamate also provoked an elevation in TNF-α production in astrocyte cultures (Figure 7B). The effect of glutamate on astrocytes was similar to that obtained with 0.5 ng/ml LPS. Glutamate had, however, no impact on IL-1β levels (data not shown). In microglial cultures, glutamate had no significant impact on Iba-1 expression at 50 μM, but it significantly increased the activation marker at 500 µM (Figure 7C). Glutamate failed, however, to stimulate cytokine production in cultured microglia regardless of the concentration tested (Figure 7D).

### CPZ + CBD Prevents TNF-α Release Induced by Glutamate in Astrocyte Cultures

**FIGURE 8:**
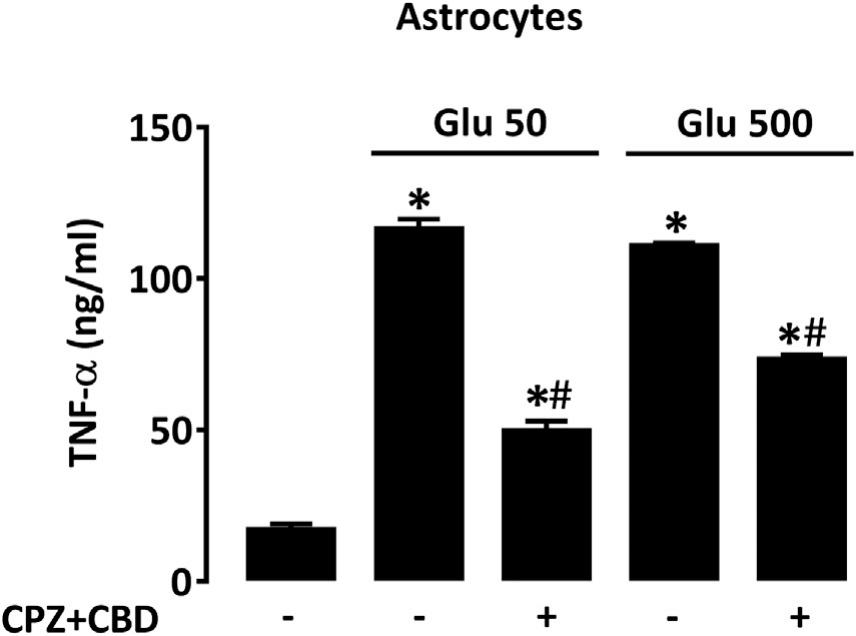
CPZ + CBD reduces TNF-α production in cultured astrocytes exposed to glutamate. Impact of CPZ (0.1 µM) + CBD (0.1 μM) on TNF-α elevation produced by glutamate (50, 500 µM) in astrocyte cultures. *p < 0.05 *vs.* control, #*p* < 0.05 *vs.* same glutamate concentration*,* n = 6; One-way ANOVA followed by Bonferroni test.

To determine to what extent TNF-α released by activated astrocytes could contribute to dyskinesia in L-DOPA-treated mice, we measured levels of this cytokine in astrocyte cultures treated with glutamate (50 and 500 µM) for 24 h in the presence or not of CPZ + CBD (both at 0.1 µM). In line with present observations in dyskinetic mice, we found that the elevation of TNF-α levels induced by glutamate in astrocyte cultures was significantly curtailed by concomitant treatment with CPZ + CBD (Figure 8).

### TNF-α-Induced Glutamate Release in Cortical Neuronal Cultures Is Prevented by Treatment With CPZ + CBD

**FIGURE 9:**
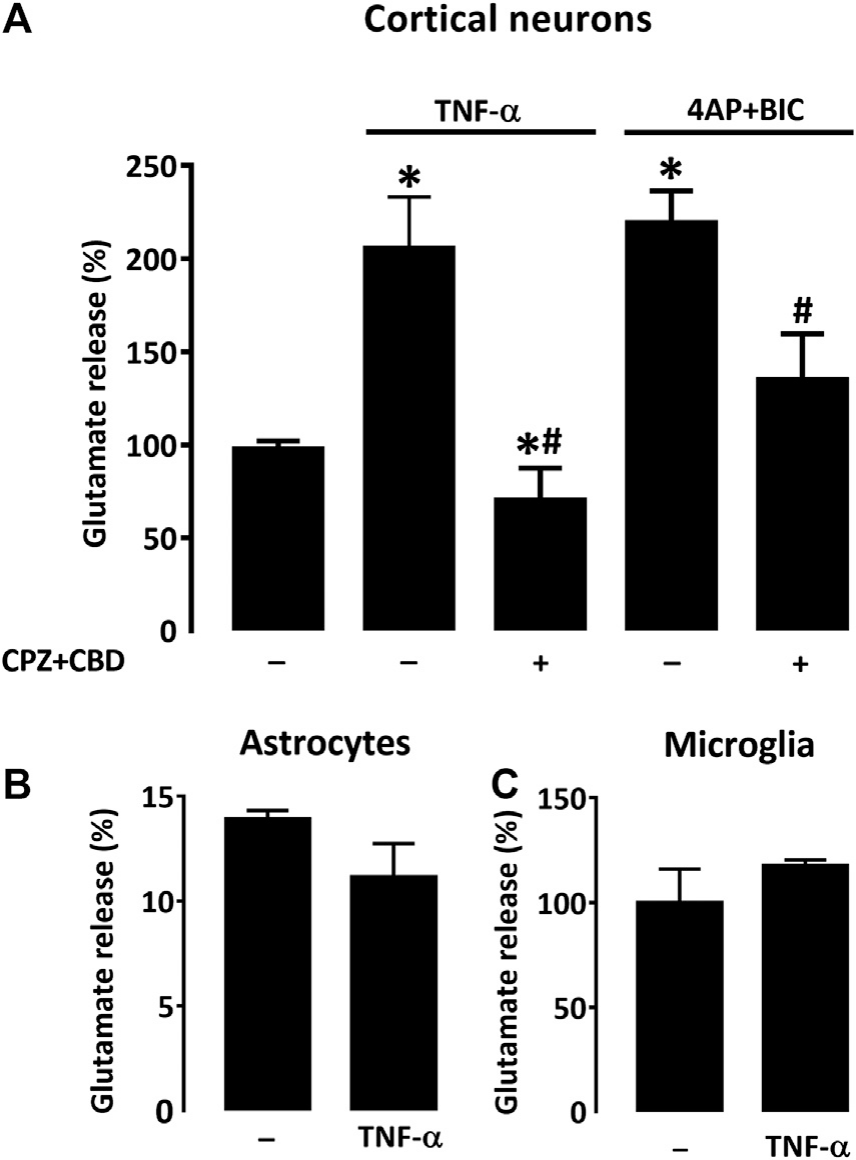
CPZ + CBD prevents the release of glutamate induced by TNF-α in cultures of cortical neurons. **(A)** Impact that a 4-days exposure to TNF-α (50 ng/ml) or the reference treatment 4AP (2.5 mM) + BIC (50 μM) exert on the release of glutamate in pure neuronal cortical cultures. A co-treatment with CPZ + CBD (both at 0.1 μM) reduced glutamate release induction in both paradigms. TNF-α failed to modulate glutamate levels in astrocyte **(B)** or microglial **(C)** cell cultures. *p < 0.05 vs. control. #*p* < 0.05 vs. TNF-α or 4-AP + BIC, n = 7; One-way ANOVA followed by Bonferroni test.

Still to better understand how TNF-α produced by astrocytes could be involved in L-DOPA-induced dyskinesia, we wished to evaluate the impact of this cytokine on the release of glutamate by cortical neurons in the presence or not of CPZ + CBD. As a model system for that, we established pure neuronal cortical cultures and exposed them to TNF-α (50 ng/ml) in the presence or not of CPZ + CBD (both at 0.1 µM). Some sets of cultures were exposed to a treatment combining 4AP (2.5 mM) + BIC (50 µM) to evoke the synaptic release of glutamate (Lavaur et al., 2016). Our results show that chronic exposure to TNF-α (96 h) resulted in a two-fold increase of glutamate release in cortical cultures (Figure 9A). Interestingly, this effect was prevented by concomitant treatment with CPZ + CBD. The treatment with 4AP + BIC also promoted glutamate release, and this effect was prevented by CPZ + CBD as well. Notably, LDH leakage assays performed on cultures treated with TNF-α and 4AP + BIC showed none of these two treatments affected neuronal survival (not shown). Despite the fact that TNF-α was reported capable of activating both microglial cells (Neniskyte et al., 2014) and astrocytes (Trindade et al., 2020) at a concentration of 50 ng/ml, we found that such a concentration had no significant impact on extracellular glutamate levels in astrocyte (Figure 9B) and microglial cell cultures (Figure 9C), suggesting that the modulatory effect of TNF-α on glutamate levels is restricted to neuronal cells in the current setting.

## Discussion

In the present study, we confirmed that the induction of dyskinesia by L-DOPA in 6-OHDA hemiparkinsonian mice is accompanied by a strong glial inflammatory response, restricted to the dorsal part of the striatum, which is depleted in dopamine. The activation of astrocytes was particularly prominent within this striatal area, and LID severity appeared correlated to increased levels of TNF-α and IL-1β in this structure. A treatment with CPZ + CBD not only efficiently reduced LID development in dyskinetic mice but also selectively decreased striatal TNF-α levels without, however, impacting other striatal inflammation markers, including IL-1β. *In vitro* studies revealed that TNF-α might originate from reactive astrocytes in response to elevated glutamate levels. Of interest, the treatment with CPZ + CBD reduced TNF-α production in astrocyte cultures challenged with glutamate. The same treatment also inhibited glutamate release induced by TNF-α in cortical neuronal cultures, leading to the hypothesis that LID may result from a pathological process in which this pro-inflammatory cytokine plays a crucial role.

### LID Occurrence in Partially Lesioned Hemiparkinsonian Mice

We established a partially lesioned hemiparkinsonian mouse model to study the cellular and molecular mechanisms underlying LID onset. In coherence with our previous work (Dos-Santos-Pereira et al., 2016) and data reported earlier (Pavon et al., 2006) we showed, here, that cellular and behavioral supersensitivity to L-DOPA develop in partially lesioned mice when residual dopamine innervation is below a critical, threshold value (Winkler et al., 2002; Feng et al., 2019). However, at variance to present results in dyskinetic mice, abnormal involuntary movements were reported to be rare or nonexistent in rats having more than 20% of their residual nigrostriatal dopaminergic innervation (Winkler et al., 2002).

### Changes in Glial Cell Morphology and Density in Hemiparkinsonian Mice in Response to L-DOPA Treatment

Glial cells are emerging as key contributors to the neuro-immune response that characterizes various neurodegenerative pathologies and neurological symptoms, including LID (Del-Bel et al., 2016; Junior et al., 2020). Notably, the L-DOPA dyskinesiogenic treatment was reported to cause a robust activation of glial cells in the striatum of hemiparkinsonian rats (Bortolanza et al., 2015a; Ramirez-Garcia et al., 2015; Del-Bel et al., 2016; Mulas et al., 2016; Carta et al., 2017). Present observations in our dyskinetic hemiparkinsonian mouse model corroborate these data and confirm the presence of activated astrocytes and microglia in the dorsal part of the striatum depleted in dopaminergic nerve fibers. Precisely, we noted that the GFAP immunosignal was globally elevated in the dorsal striatal area following L-DOPA treatment in comparison to vehicule-treated animals. This resulted from an augmentation of GFAP^+^ cells, an increased volume of astrocytic soma and an enlargement of primary processes. The number of Iba-1^+^ microglial cells was also explicitly increased in the dorsal striatal region, with a high proportion of these cells having a reactive morphology characterized by a larger amoeboid-like soma with short processes or a smaller soma with thick processes. Note that the strong astrocyte and microglial responses, observed 21 days after 6-OHDA lesioning, became either moderate or absent, respectively, in Veh-treated animals, which were analyzed 42 days after stereotaxic surgery. This is in good correlation to previous observations (Henry et al., 2009; Stott and Barker, 2014) and confirms that L-DOPA administration is a primary requirement for glial cell activation in dyskinetic mice.

### The Glial Cell Response in Dyskinetic Mice Is Associated With Elevations of TNF-α and IL-1β in the Dopamine-Denervated Striatum

The activation of astrocytes and microglial cells in dyskinetic mice was correlated to increased levels of the two pro-inflammatory cytokines TNF-α and IL-1β in the dorsal injured striatum. Related to our current observations, Barnum and colleagues (Barnum et al., 2008) also described an elevation of IL-1β levels and a trend for increased levels of TNF-α in the lesioned striatum of dyskinetic rats. In 6-OHDA-lesioned hemiparkinsonian rats, Mulas, and colleagues (Mulas et al., 2016) reported that LID was associated with the production of TNF-α by microglial cells. Even if we did not identify the subtype of cells producing these two cytokines in the lesioned striatum of dyskinetic mice, they were likely produced by activated glial cells even if we cannot exclude a neuronal origin. Overall, these *in vivo* data confirm the idea that in case of dopaminergic degeneration, a necessary condition for LID onset and perpetuation is the occurrence of a permissive pro-inflammatory environment resulting from glial cell activation (Del-Bel et al., 2016; Carta et al., 2017; Pisanu et al., 2018).

### The Antidyskinetic Treatment With Capsazepine + Cannabidiol Decreases TNF-α but Not IL-1β Levels in L-DOPA Treated Dyskinetic Mice

We recently reported that the non-psychoactive cannabinoid CBD exerts an antidyskinetic effect in L-DOPA-treated mice when used in combination with the vanilloid TRPV1 channel inhibitor, CPZ (Dos-Santos-Pereira et al., 2016). Here, we found that this treatment significantly decreased LID intensity scores by at least 40%. The CPZ + CBD treatment also reduced TNF-α levels in the dorsal striatum of dyskinetic mice, indicating that this cytokine may participate in LID development. At variance, the CPZ + CBD combination failed to affect IL-1β levels, which indicates that this treatment was effective on a glial mechanism that might precisely control TNF-α expression.

In line with this, TNF-α synthesis is controlled by an NF-κB-dependent mechanism in glial cells (Olmos and Llado, 2014; Dos-Santos-Pereira et al., 2020), whereas IL-1β secretion is modulated through activation of the NLRP3 inflammasome complex (He et al., 2016). Even if we cannot conclude on a possible implication of IL-1β in LID based on present data, a previous study has demonstrated that abnormal involuntary movements were reducible by intrastriatal injection of an IL-1β receptor antagonist (Barnum et al., 2008), suggesting that both TNF-α and IL-1β may contribute to LID development. Note also that CPZ + CBD did not reduce the expression of GFAP in activated astrocytes, indicating that the inhibitory effect of its antidyskinetic treatment was presumably restricted to signaling events controlling TNF-α expression.

### Nature of the Signal That Triggers the Glial Inflammatory Response in Dyskinetic Mice

To study the nature of the inflammogens possibly at the origin of the glial response in L-DOPA-treated dyskinetic mice, we established purified cultures of mouse astrocytes and microglial cells and exposed them to candidate molecules possibly involved in this process. We first tested dopamine and its precursor L-DOPA. Indeed, LID results primarily from excessive dopamine levels in the striatum as a consequence of the pulsatile nature of the stimulation with L-DOPA (Meissner et al., 2006; Mulas et al., 2016). Western immunoblotting assessment of GFAP expression in astrocyte cultures revealed that neither L-DOPA nor dopamine could increase expression levels of this protein at the two concentrations tested. Likewise, the inflammation marker Iba-1 was not augmented when microglial cells were challenged with L-DOPA or dopamine. We even observed that the highest test concentration of dopamine resulted in reduced basal expression of Iba-1, in agreement with reports showing that the neurotransmitter can exert immunosuppressive effects (Yan et al., 2015; Dos-Santos-Pereira et al., 2020). This may signify that the depletion in dopamine may create itself permissive conditions for inflammation-type reactions in dyskinesia.

It is also known that glutamate is crucially involved in the onset of LID: 1) microdialysis assessment revealed that basal extracellular glutamate levels are substantially increased in dyskinetic rodent models (Jonkers et al., 2002; Robelet et al., 2004), even if this is still debated (Nevalainen et al., 2013); 2) corticostriatal glutamatergic synapses are overactivated during L-DOPA-induced dyskinesia (LID) (Morin and Di Paolo, 2014; Mellone and Gardoni, 2018); 3) LID can be inhibited by several antagonists of NMDA glutamate receptors (Hadj Tahar et al., 2004; Baufreton et al., 2018), including amantadine the only approved drug for LID treatment (Perez-Lloret and Rascol, 2018), even though another mechanism probably involving Kir2 K^+^ channel inhibition was proposed for this compound (Shen et al., 2020) and 4) LID reduction can be achieved by activation of DREAM, a protein that negatively regulates NMDA receptors (Ruiz-DeDiego et al., 2015).

Therefore, we evaluated the response of astrocytes and microglial cells to glutamate. We found that 50 and 500 µM glutamate caused a robust increase of the GFAP immunosignal in astrocytes in good agreement with previous observations (Romao et al., 2008). Of interest, this response was associated with a robust elevation of TNF-α levels, whereas IL-1β remained unchanged. Glutamate also provoked an increased expression of the activation marker Iba-1 in microglial cultures, but this increase reached significance only at the highest test concentration. Besides, neither TNF-α nor IL-1β increased in microglial cultures exposed to glutamate regardless of the neurotransmitter’s concentration. So, our current data suggest that astrocyte (but not microglia) activation might result from elevated striatal glutamate levels in dyskinetic mice.

Because LID and striatal TNF-α levels were abated in dyskinetic mice that received the antidyskinetic treatment with CPZ + CBD, we tested the impact this treatment could have on TNF-α release induced by glutamate in astrocyte cultures. Adding support to the idea that elevated glutamate could contribute to astrocyte activation during LID development, we found that CPZ + CBD efficiently counteracted the elevation of TNF-α in glutamate-treated astrocytes. This suggests that TNF-α might be produced in the denervated striatum of dyskinetic mice not only by microglial cells, as suggested before (Mulas et al., 2016), but also by activated astrocytes.

### Potential Consequences of a Rise in TNF-α in L-DOPA-Treated Dyskinetic Mice

TNF-α can modify neuronal network excitability in various brain areas, including the striatum. Therefore, we aimed to determine whether TNF-α produced by glutamate-activated astrocytes could reinforce glutamate release from corticostriatal inputs. Coherent with previous observations, we found that chronic exposure to TNF-α stimulated glutamate release in cortical neuronal cultures. The fact that glutamate excitotoxicity was not observable in cortical cultures exposed to TNF-α (not shown) indicated that glutamate release induced by this cytokine was essentially synaptic. Indeed, neuronal death by excitotoxicity is primarily due to glutamate extrasynaptic release (Lavaur et al., 2016).

**FIGURE 10:**
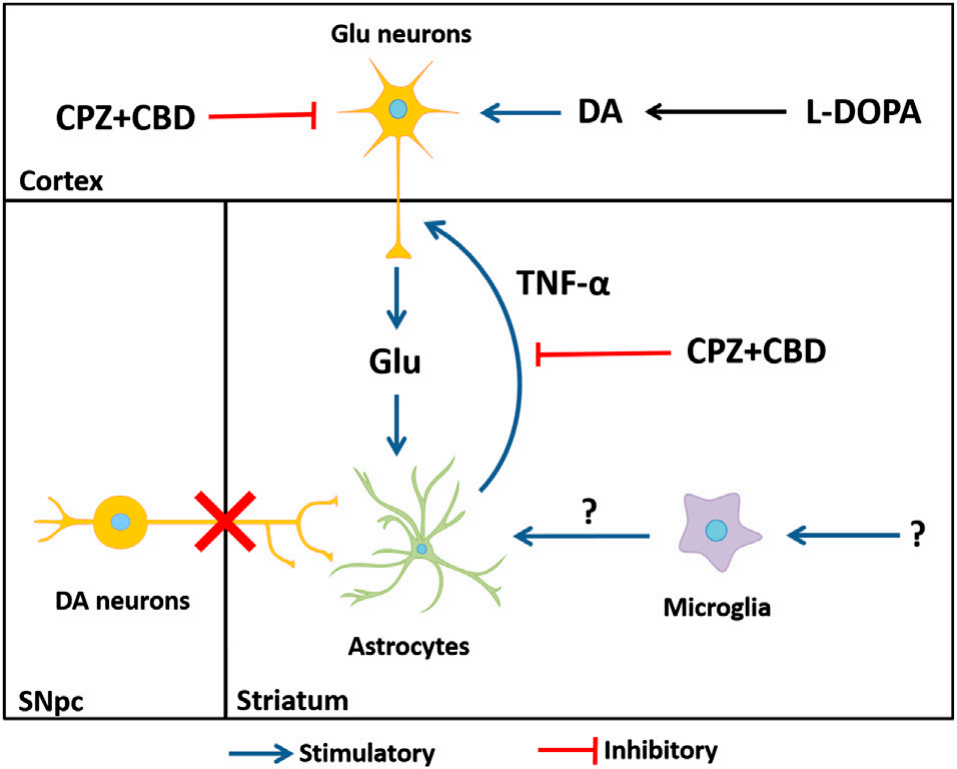
Hypothetical mechanisms through which astrocytes, glutamate, and TNF-α might contribute to LID onset and perpetuation. The dyskinesiogenic treatment with L-DOPA causes the stimulation of corticostriatal inputs, elevating glutamate levels at corticostriatal synapses. The elevation of glutamate elicits an inflammatory-type response in astrocytes. Reactive astrocytes release the pro-inflammatory cytokine TNF-α, which is capable of further enhancing the release of glutamate by cortical neurons in a self-reinforcing manner, thus creating a vicious circle that contributes to LID perpetuation. Note that glutamate might be also released by activated microglial cells through the activation of the Xc^−^ antiporter system (Farber and Kettenmann, 2005). The elevation of striatal IL1-β levels in dyskinetic mice appears unrelated to glutamate release. DA: dopamine; Glu: glutamate; SNpc: substantia nigra pars compacta.

Most interestingly, glutamate release induced by TNF-α in cortical neurons was inhibited by CPZ + CBD, indicating that the antidyskinetic treatment may operate not only by repressing glutamate-activated astrocytes but also by inhibiting the stimulatory effect of TNF-α at corticostriatal synapses. Based on current results, we cannot exclude the fact that astrocyte activation could also partly result from glutamate released by activated microglial cells through the activation of the Xc^−^ antiporter system (Farber and Kettenmann, 2005). However, inflammatory cues at the origin of microglial cell activation remain to be identified in this context. A schematic drawing that summarizes how inflammatory-type mechanisms may contribute to LID development is given in figure 10

To conclude, we are well aware that the culture systems used in this study cannot fully recapitulate the complex and multifaceted nature of dyskinesia. Yet, by combining *in vitro* and *in vivo* approaches, we confirmed in this study that glial inflammatory processes might be crucially involved in the development and perpetuation of LID through mechanisms that involve TNF-α. In particular, we suggest that TNF-α produced by glutamate-activated astrocytes may contribute to LID by exacerbating the excitability of corticostriatal inputs. Because multimodal approaches may be necessary to suppress LID, one may assume that treatments targeting glial-dependent production of TNF-α may represent an attractive add-on therapy to other antidyskinetic treatments.

## Data Availability Statement

The raw data supporting the conclusions of this article will be made available by the authors, without undue reservation.

## Ethics Statement

Protocols for animal studies were reviewed and approved in Brazil by Comitê de Ética na Utilização de Animais (CEUA 026/2013) and in France by Committee on the Ethics of Animal Experiments Charles Darwin n°5.

## Author Contributions

MP: Conceptualization, Investigation, Experimental research, Formal Analysis, Writing: Original Draft Preparation, Review and Editing GA, JR, SH: Experimental research, Formal Analysis RR-V: Funding acquisition; Project administration, Supervision, Review and Editing PM, EBEDB: Conceptualization, Investigation, Funding acquisition, Project administration, Supervision, Validation, Writing: Review and Editing.

## Funding

This project was carried out in the framework of the CAPES-COFECUB program between the French and Brazilian research institutions (project 88887.192409/2018-00 - #Me928/19). Financial support was provided by following Brazilian agencies - Coordenação de Aperfeiçoamento de Pessoal de Nível Superior (CAPES; PROEX0051047 - USP/RP), Fundação de Amparo à Pesquisa do Estado de São Paulo (FAPESP; 2014/25029-4; 2017/24304-0; 2017/14207-7; 2018/03482-0) and Conselho Nacional de Desenvolvimento Cientifico e Tecnológico (CNPq; 201187/2016-7). This work also benefited from support by Investissements d’Avenir (ANR-10-IAIHU-06) and the Translational Research Infrastructure for Biotherapies in Neurosciences (ANR-11-INBS-0011-NeurATRIS).

## Conflict of Interest

The authors declare that the research was conducted in the absence of any commercial or financial relationships that could be construed as a potential conflict of interest.

## Abbreviations

6-OHDA, 6-hydroxydopamine; AIMs, abnormal involuntary movements; CBD, cannabidiol; COX-2, cyclooxygenase 2; CPZ, capsazepine; DA, dopamine; FCS, fetal calf serum; GAPDH, glyceraldehyde 3-phosphate dehydrogenase; GFAP, Glial Fibrillary acidic protein; Iba-1, ionized calcium-binding adaptor molecule-1; IL-1β, interleukin-1β; ir, immunoreactive; L-DOPA, l-3,4-dihydroxyphenylalanine; LID: L-DOPA-induced dyskinesia; LPS, lipopolysaccharide; NOS, nitric oxide synthase; PBS, Dulbecco's phosphate-buffered saline medium; PEI, polyethyleneimine; TH, tyrosine hydroxylase; TNF-α, tumor necrosis factor-α; TRPV-1: Transient receptor potential vanilloid-1; Veh, vehicle.
