# Supplementary material for: Contributive Role of TNF-α to L-DOPA-Induced Dyskinesia in a Unilateral 6-OHDA Lesion Model of Parkinson’s Disease
Source: Front Pharmacol. 2021 Jan 11;11:617085. doi: 10.3389/fphar.2020.617085 (PMC7836015; doi:10.3389/fphar.2020.617085)
Supplement: Supplementary file 1 [file table1.doc]

**Supplementary Table –** Statistical analysis performed in this study.

- Group characteristics are properly identified throughout the text or in figure 1
- The number of individual assets used in each group is described in figure legends

| **Figure** | **Parameter** | **Test** | **Value** |
| --- | --- | --- | --- |
| **Figure 2A** | Axial, Limb and Orofacial abnormal involuntary movements score  *Groups: sham vs. 6-OHDA vs. Veh vs. L-DOPA* | Kruskal-Wallis followed by Dunn’s multiple comparison’s test | **X²(3) = 26.28**  **p < 0.0001** |
| **Figure 2B** | Rotational behavior score  *Groups: sham vs. 6-OHDA vs. Veh vs. L-DOPA* | Kruskal-Wallis followed by Dunn’s multiple comparison’s test | **X²(3) = 13.4**  **p = 0.0038** |
| **Figure 2C** | Number of TH+ neurons in substantia nigra pars compacta/0.5mm²  *Groups: sham vs. 6-OHDA vs. Veh vs. L-DOPA* | Ordinary One-way ANOVA followed by Bonferroni’s test | **F_3,24_ = 295.4**  **p < 0.0001** |
| **Figure 2D** | Integrated optical density of TH+ fibers in striatum  *Groups: sham vs. 6-OHDA vs. Veh vs. L-DOPA* | Ordinary One-way ANOVA followed by Bonferroni’s test | **F_3,24_ = 190.6**  **p < 0.0001** |
| **Figure 3A** | Integrated optical density of GFAP+ cells in dorsal striatum  *Groups: sham vs. 6-OHDA vs. Veh vs. L-DOPA* | Ordinary One-way ANOVA followed by Bonferroni’s test | **F_3,48_ = 26.12**  **p < 0.0001** |
| **Figure 3B** | Number of activated Iba-1+ cells in dorsal striatum/0.5mm²  *Groups: sham vs. 6-OHDA vs. Veh vs. L-DOPA* | Ordinary One-way ANOVA followed by Bonferroni’s test | **F_3,48_ = 16.33**  **p < 0.0001** |
| **Figure 4A** | Striatal TNF-α concentration (pg/mg of protein)  *Groups: sham vs. Veh vs. L-DOPA* | Ordinary One-way ANOVA followed by Bonferroni’s test | **F_2,15_ = 20.37**  **p < 0.0001** |
| **Figure 4B** | Striatal IL-1β concentration (pg/mg of protein)  *Groups: sham vs. Veh vs. L-DOPA* | Ordinary One-way ANOVA followed by Bonferroni’s test | **F_2,15_ = 14.33**  **p = 0.0003** |
| **Figure 4C** | Correlation of striatal TNF-α concentration and abnormal involuntary movements score | Spearman correlation | **r = 0.98**  **p = 0.005** |
| **Figure 4D** | Correlation of striatal IL-1β concentration and abnormal involuntary movements score | Spearman correlation | **r = 0.94**  **p = 0.01** |
| **Figure 5A** | Axial, Limb and Orofacial abnormal involuntary movements score  *Groups: Veh/L-DOPA vs. CPZ+CBD/L-DOPA* | Mann Whitney test | **U = 0**  **p = 0.0022** |
| **Figure 5B** | Striatal TNF-α concentration (pg/mg of protein)  *Groups: Veh/L-DOPA vs. CPZ+CBD/L-DOPA* | Unpaired student’s t-test | **t = 4.377**  **df = 10**  **p = 0.0014** |
| **Figure 5C** | Striatal IL-1β concentration (pg/mg of protein)  *Groups: Veh/L-DOPA vs. CPZ+CBD/L-DOPA* | Unpaired student’s t-test | **t = 1.89**  **df = 10**  **p = 0.0888** |
| **Figure 5D** | Integrated optical density of GFAP+ cells in dorsal striatum  *Groups: Veh/L-DOPA vs. CPZ+CBD/L-DOPA* | Unpaired student’s t-test | **t = 0.05**  **df = 14**  **p = 0.9619** |
| **Figure 5E** | Number of activated Iba-1+ cells in dorsal striatum/0.5mm²  *Groups: Veh/L-DOPA vs. CPZ+CBD/L-DOPA* | Unpaired student’s t-test | **t = 0.18**  **df = 14**  **p = 0.8576** |
| **Figure 6A** | GFAP expression in primary cultures of astrocytes (arbitrary units)  *Groups: Control vs. 3 µM L-DOPA vs. 10 µM L-DOPA* | Ordinary One-way ANOVA followed by Bonferroni’s test | **F_2,9_ = 1.324**  **p = 0.3134** |
| **Figure 6B** | GFAP expression in primary cultures of astrocytes (arbitrary units)  *Groups: Control vs. 3 µM L-DA vs. 10 µM L-DA* | Ordinary One-way ANOVA followed by Bonferroni’s test | **F_2,9_ = 1.896**  **p = 0.2056** |
| **Figure 6C** | Iba-1 expression in primary cultures of microglial cells (arbitrary units)  *Groups: Control vs. 3 µM L-DOPA vs. 10 µM L-DOPA* | Ordinary One-way ANOVA followed by Bonferroni’s test | **F_2,9_ = 2.04**  **p = 0.1860** |
| **Figure 6D** | Iba-1 expression in primary cultures of microglial cells (arbitrary units)  *Groups: Control vs. 3 µM L-DA vs. 10 µM L-DA* | Ordinary One-way ANOVA followed by Bonferroni’s test | **F_2,9_ = 8.292**  **p = 0.0091** |
| **Figure 7A** | GFAP expression in primary cultures of astrocytes (arbitrary units)  *Groups: Control vs. 50 µM Glu vs. 500 µM Glu* | Ordinary One-way ANOVA followed by Bonferroni’s test | **F_2,9_ = 79.03**  **p < 0.0001** |
| **Figure 7B** | TNF-α concentration produced and released by primary cultures of astrocytes (mg/ml)  *Groups: Control vs. 50 µM Glu vs. 500 µM Glu vs. LPS 0.5 ng/ml* | Ordinary One-way ANOVA followed by Bonferroni’s test | **F_3,20_ = 144**  **p < 0.0001** |
| **Figure 7C** | Iba-1 expression in primary cultures of microglial cells (arbitrary units)  *Groups: Control vs. 50 µM Glu vs. 500 µM Glu* | Ordinary One-way ANOVA followed by Bonferroni’s test | **F_2,9_ = 74.72**  **p < 0.0001** |
| **Figure 7D** | TNF-α concentration produced and released by primary cultures of microglial cells (mg/ml)  *Groups: Control vs. 50 µM Glu vs. 500 µM Glu vs. LPS 0.5 ng/ml* | Ordinary One-way ANOVA followed by Bonferroni’s test | **F_3,20_ = 342.1**  **p < 0.0001** |
| **Figure 8** | TNF-α concentration produced and released by primary cultures of microglial cells (mg/ml)  *Groups: Control vs. 50 µM Glu vs. CPZ/CBD + 50 µM Glu vs. 500 µM Glu vs. CPZ/CBD + 500 µM Glu* | Ordinary One-way ANOVA followed by Bonferroni’s test | **F_4,15_ = 338.5**  **p < 0.0001** |
| **Figure 9A** | Glutamate release by primary cultures of cortical neurons (% of non-treated cells)  *Groups: Control vs. TNF-α vs. CPZ/CBD + TNF-α vs. 4AP/BIC vs. CPZ/CBD + 4AP/BIC* | Ordinary One-way ANOVA followed by Bonferroni’s test | **F_4,30_ = 10.72**  **p < 0.0001** |
| **Figure 9B** | Glutamate release by primary cultures of astrocytes (% of non-treated cells)  *Groups: Control vs. TNF-α* | Unpaired student’s t-test | **t = 1.376**  **df = 12**  **p = 0.2063** |
| **Figure 9C** | Glutamate release by primary cultures of microglial cells (% of non-treated cells)  *Groups: Control vs. TNF-α* | Unpaired student’s t-test | **t = 1.092**  **df = 12**  **p = 0.3360** |
